# Proteins Involved in Plant Dehydration Protection: The Late Embryogenesis Abundant Family

**DOI:** 10.3390/biom12101380

**Published:** 2022-09-27

**Authors:** Steffen P. Graether

**Affiliations:** 1 Department of Molecular and Cellular Biology, University of Guelph, Guelph, ON N1G 2W1, Canada; graether@uoguelph.ca; 2 Graduate Program in Bioinformatics, University of Guelph, Guelph, ON N1G 2W1, Canada

Plants have evolved a number of different ways to deal with different types of abiotic stresses; at the molecular level, dehydration can cause multiple forms of damage to different biomolecules [1]. With proteins, the most prominent form of damage is denaturation due to the lack of water, which is a key molecule that drives protein folding, while with membranes, changes in water content and/or the lowering of the temperature can decrease membrane fluidity, a key property in maintaining proper membrane function. Nucleic acids may be more structurally tolerant to dehydration [2], but dehydration can also lead to the formation of reactive oxygen species (ROS), which can cause strand breakage. Plants express a number of different proteins to reduce the amount of damage caused by dehydration stress (drought, cold, salinity), where some of the best characterized members of the dehydration protein family are the late embryogenesis abundant (LEA) proteins [3]. Originally described in seeds, LEA proteins have since been identified in many adult plant tissues. One interesting property of nearly all LEA proteins is that they are intrinsically disordered proteins (IDPs), that is, they do not have a stable ordered structure when alone in solution.

This Special Issue of *Biomolecules* examines LEA proteins through three research articles and two reviews, focussing mainly on how these proteins may function to protect organisms from dehydration stresses. While there is considerable evidence to suggest that LEA proteins are involved in dehydration stress [3], the exact mechanism by which these proteins function in vivo is still a matter of debate. These articles and reviews highlight the characterization of several LEA proteins, both in plants and in one of the few animals in which LEA proteins are found, by examining their different sequence, structure, and function relationships, leading the way to more complex experiments that will answer how LEA proteins function in vivo.

Some IDPs can undergo a process known as liquid–liquid phase separation (LLPS) [4]. These proteins are able to weakly associate with themselves or RNA, resulting in the formation of a membraneless organelle. Ginsawaeng et al. [5] studied several LEA proteins from *Arabidopsis thaliana* using a combination of bioinformatics and fluorescence microscopy to examine the localization and phase separation properties of these proteins. In terms of self-association, they found that some LEA proteins could form oligomers, and, most interestingly, LEA proteins LEA9 and LEA48 formed droplet structures, showing that they form LLPS condensates. The authors suggest that the formation of these condensates may play a role in the stress response as a sensor.

While mainly found in plants, some LEA proteins have also been described in animals, including the LEA_5 protein in *Artemia franciscana*. The paper by Janis et al. [6] examined the structure and function of the *Af*LEA1 protein, and the formation of LLPS both in solution and in *Drosophila melanogaster* cells. They found that under hydrated conditions, the secondary structure of *Af*LEA1 was mostly a mix of random coil and β-strands, but when dried the protein gained significant helical content. Bioinformatic analysis suggests that the protein has a high probability to form a condensate, a prediction which was demonstrated to be correct; at low water concentrations or in the presence of RNA, *Af*LEA1 underwent LLPS formation. In addition, the expression of *Af*LEA1 in Drosophila Kc167 cells showed not only that the protein was able to form LLPS condensates in cells, but also that the cells were better able to maintain cytoplasmic viscosity even under desiccation conditions. The authors propose that the LLPS properties and ability of *Af*LEA1 to have multiple modes of dehydration protection could be a feature of other LEA_5 proteins.

Dehydrins, well-characterized members of the LEA protein family, have been shown to be overexpressed during dehydration, including high salt stress. The paper by Ghanmi et al. compares the dehydrin sequences from 10 halophytic plants and the overexpression of dehydrins in three well-characterized halophytes [7]. Dehydrins consist of several different protein architectures, where each architecture contains a different combination of sequence motifs. The authors found that the distribution of these architectures in halophytes was different to that in glycophytes, suggesting that certain architectures may be better tuned to protect plants from specific stresses. Sequence conservation in the motifs was largely the same between halophytes and glycophytes, though the researchers did find that charged amino acids were more strongly conserved in halophytes. Lastly, they also examined the expression of dehydrins during salt stress. Similar to the sequence analysis, they found that certain dehydrin architectures were more highly expressed during salt stress compared to others.

This Special Issue also includes two reviews on dehydrins by two different research groups. The review by Aziz et al. [8] examines dehydrins with an emphasis on plant physiology, and includes a description of dehydrins in *Phoenix dactylifera* as an example of an extremophile plant. As an overview, they list all of the tissue types in which dehydrins are found, and what abiotic stresses cause different expression levels of dehydrins. The authors discussed several genome-wide association studies (GWAS), with the reasoning that this will be important in the selection of LEA genes to create more stress-resistant crops. The researchers complete the review with a detailed summary of the many in vitro assays that have been used to characterize dehydrin function.

The review by Smith and Graether [9] covers several similar topics to the previous review, but more strongly emphasizes the biochemical aspects of dehydrins. The authors note that the K-segment, often used to define whether a gene encodes a dehydrin, was better captured by a regular expression than a simple protein sequence. They then continue to discuss the other conserved motifs as regular expressions, and suggest that dehydrins should be defined by seven different architectures rather than the previously defined five. The review continues with analyses of recent studies on the cryoprotection assays and mechanisms of dehydrins protecting membranes, protein, and DNA from damage caused by cold stress.
